# Modelling the effectiveness of surveillance based on metagenomics in detecting, monitoring, and forecasting antimicrobial resistance in livestock production under economic constraints

**DOI:** 10.1038/s41598-023-47754-w

**Published:** 2023-11-21

**Authors:** Ofosuhene O. Apenteng, Frank M. Aarestrup, Håkan Vigre

**Affiliations:** 1 Research Group for Genomic Epidemiology, National Food Institute, Technical University of Denmark, Kongens Lyngby, Denmark; 2 Section of Animal Welfare and Disease Control, Department of Veterinary and Animal Sciences, University of Copenhagen, Copenhagen, Denmark

**Keywords:** Computational biology and bioinformatics, Genetics, Mathematics and computing

## Abstract

Current surveillance of antimicrobial resistance (AMR) is mostly based on testing indicator bacteria using minimum inhibitory concentration (MIC) panels. Metagenomics has the potential to identify all known antimicrobial resistant genes (ARGs) in complex samples and thereby detect changes in the occurrence earlier. Here, we simulate the results of an AMR surveillance program based on metagenomics in the Danish pig population. We modelled both an increase in the occurrence of ARGs and an introduction of a new ARG in a few farms and the subsequent spread to the entire population. To make the simulation realistic, the total cost of the surveillance was constrained, and the sampling schedule was set at one pool per month with 5, 20, 50, or 100 samples. Our simulations demonstrate that a pool of 20–50 samples and a sequencing depth of 250 million fragments resulted in the shortest time to detection in both scenarios, with a time delay to detection of change of $>\hspace{0.17em}$15 months in all scenarios. Compared with culture-based surveillance, our simulation indicates that there are neither significant reductions nor increases in time to detect a change using metagenomics. The benefit of metagenomics is that it is possible to monitor all known resistance in one sampling and laboratory procedure in contrast to the current monitoring that is based on the phenotypic characterisation of selected indicator bacterial species. Therefore, overall changes in AMR in a population will be detected earlier using metagenomics due to the fact that the resistance gene does not have to be transferred to and expressed by an indicator bacteria before it is possible to detect.

## Introduction

The ability to implement targeted control measures in due time against emerging antimicrobial resistance (AMR) depends on an efficient monitoring system of AMR in humans, animals, and the environment^1–6^. In Denmark, a surveillance programme for antimicrobial usage and AMR (DANMAP) was established in 1995^7^. Since its establishment, DANMAP has been based on phenotypic-based characterisation of clinical isolates of selected pathogens and selected indicator bacteria from samples in animals, humans, and food using minimum inhibitory concentration (MIC) panels^7,8^. Antimicrobial resistance genes (ARGs) can occur in all bacterial species^9,10^. Using only a few selected bacteria for detecting the emerging or increased occurrence of AMR might miss the emergence and spread of AMR in the entire resistome^7,8,11^. With the ongoing development of gene sequencing technology, it is now possible to investigate the presence and abundance of ARGs in a sample using shotgun metagenomic sequencing of DNA fragments extracted from all organisms in the sample, and subsequently mapping obtained sequences of nucleobases against databases with known sequences of nucleobases for ARGs^11–16^. It has been shown that the metagenomics approach is a useful and reliable measurement for AMR in pig production^17–21^, and we have recently shown the added value of the metagenomics approach in direct comparison to the phenotypic surveillance used in the Danish DANMAP program^22^. It has also been suggested that the use of metagenomics will increase the likelihood that emerging ARGs are detected earlier since the whole microbiota in the sample is being analysed for AMR^23–25^.

However, the likelihood of detecting an emerging or significant quantitative change of an ARG in a population using metagenomics^26^ depends, in addition to the actual occurrence of the ARG, on the design of the monitoring system. This includes a fraction of the population of interest where samples are collected (sample size), the frequency of sampling, and how much of the physical sample is investigated [in metagenomics, this is equivalent to how many extracted DNA fragments are sequenced (sequencing depth)].

For the national surveillance of AMR in Denmark and many other countries, a key performance parameter is the lag time between the de novo emergence of a novel ARG in a population or a real change in the occurrence of an ARG already present and when this change will be detected as a significant change in the surveillance program (sensitivity). It is also of practical importance that the program does not result in too many false alarms. Thus, in situations with a stable AMR level, the program should not indicate changes in the occurrence of AMR (specificity). The sensitivity and specificity of a monitoring program are often negatively correlated.

The presented study aimed to, given economic restrictions, assess different pooling strategies for detecting emergence and the increase in the prevalence of ARGs in the Danish pig population by simulation. In the simulation, we took into account the stochasticity related to sampling and laboratory procedures and the economic restrictions. Based on the simulated data, we estimated the sensitivity and specificity to compare pooling strategies in the surveillance of ARGs in a population of production animals. In the study, we show that by using relatively large pools combined with metagenomics, it is possible to monitor the all ARGs in a population. The results of the simulations show that with the use of metagenomics, it is possible to monitor all known ARGs, both detecting changes in endemic and detecting newly introduced ARGs.

## Results

**Figure 1:**
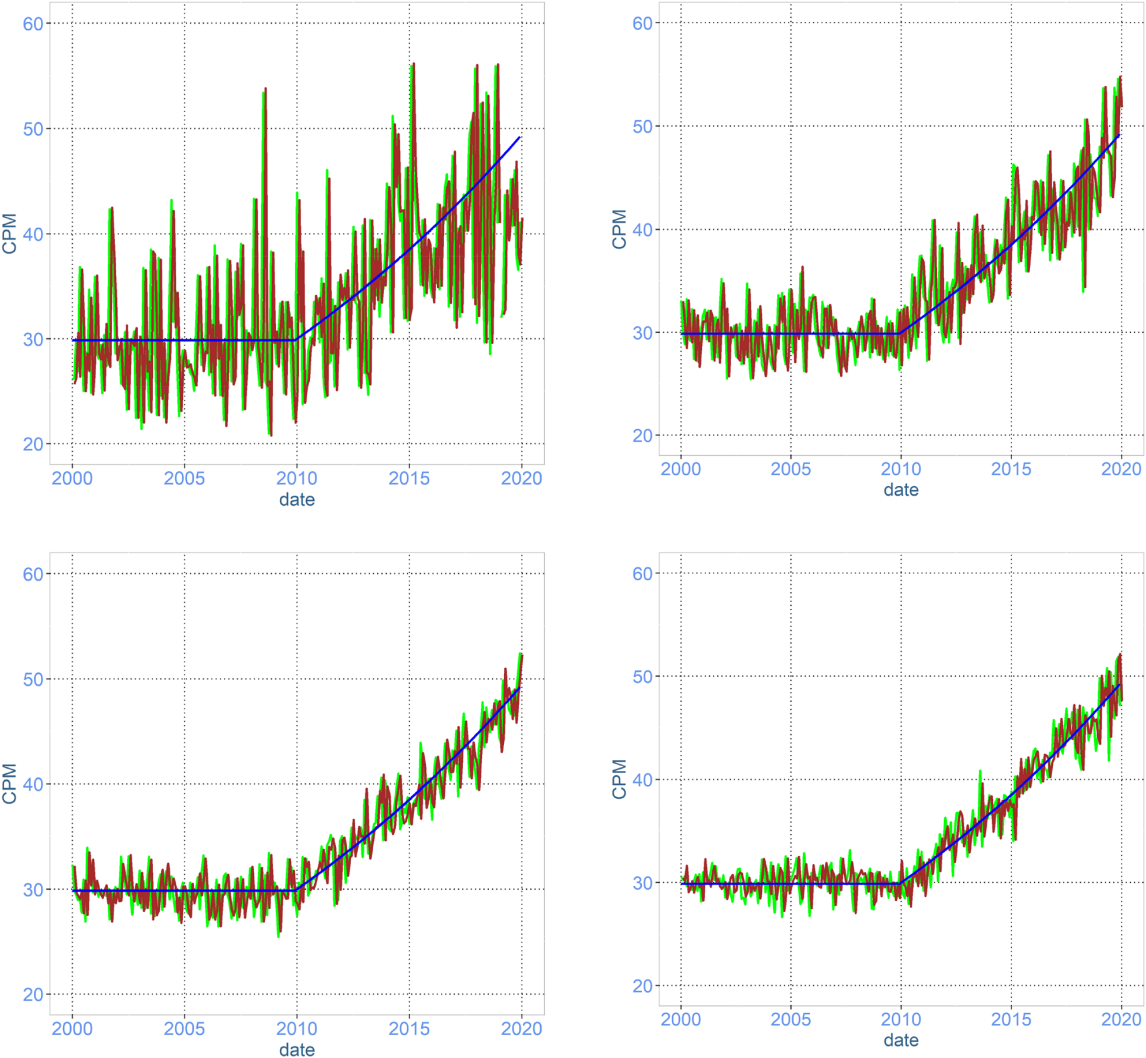
Time-series plot of simulated data representing a scenario of surveillance for 20 years of genes coding for efflux pumps causing resistance against tetracycline in swine faeces. The simulated sampling schedule is monthly collections of 5 samples (upper left), 20 samples (upper right), 50 samples (lower left), and 100 samples (lower right) of faeces from random pigs in the population. All of the samples collected per month are pooled and analysed using metagenomics. The blue line is the true CPM in the population (same in all graphs), the brown line is CPM in the pooled sample and the green line is the observed CPM in metagenomics.

**Figure 2:**
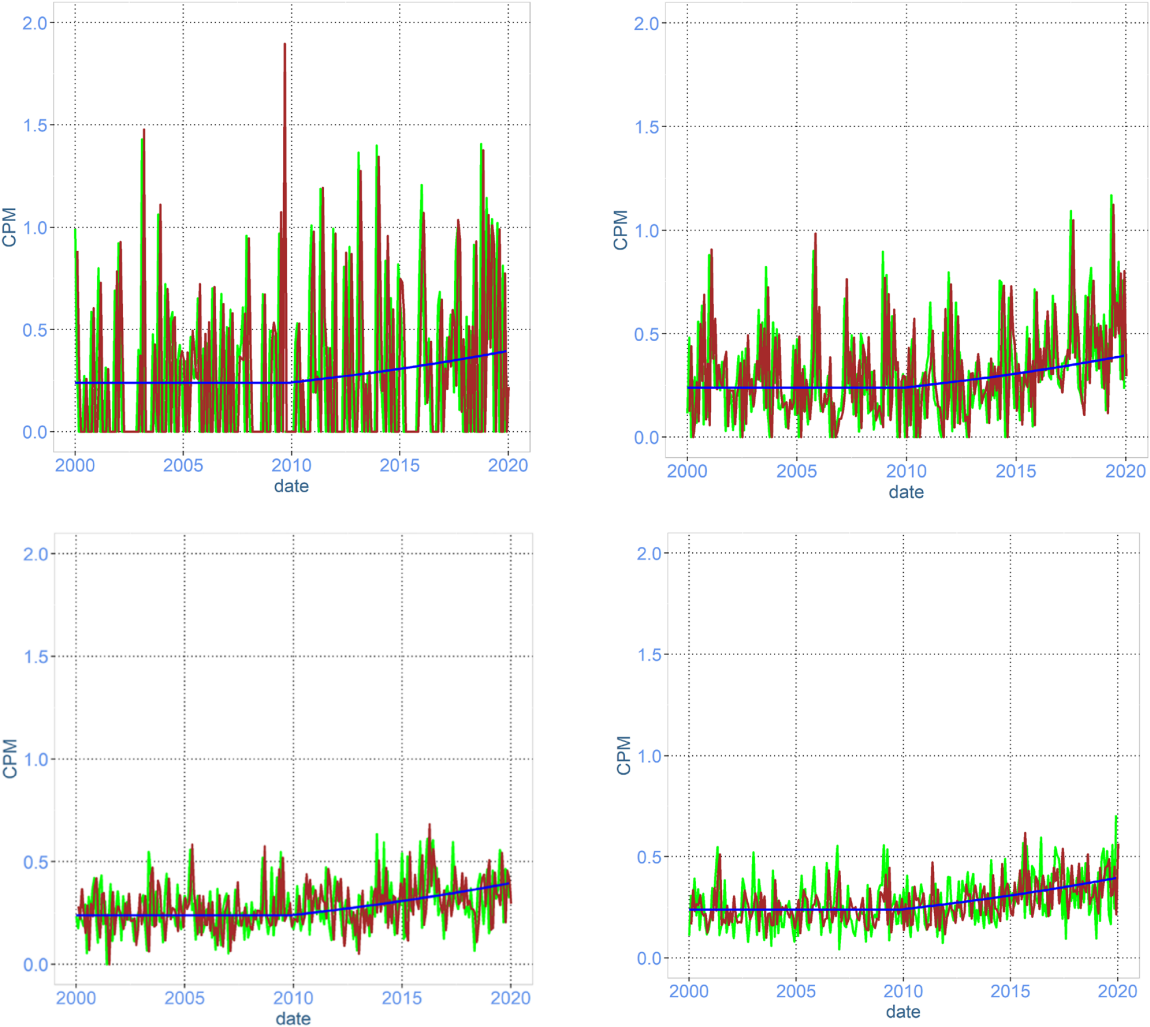
Time-series plot of simulated data representing a scenario of surveillance for 20 years of genes coding for resistance against gentamicin in swine faeces. The simulated sampling schedule is monthly collections of 5 samples (upper left), 20 samples (upper right), 50 samples (lower left), and 100 samples (lower right) of faeces from random pigs in the population. All of the samples collected per month are pooled into one technical sample and analysed using metagenomics. The blue line is the true CPM in the population (same in all graphs), the brown line is CPM in the pooled sample and the green line is the observed CPM in metagenomics.

In this study, we have measured the occurrence of ARGs as the counts per million (#CPM), which should be interpreted as how many of the sequenced gene fragments were representing a specific ARG (normalised to 1,000,000). In Fig. 1 (ARGs coding for efflux pumps causing tetracycline resistance) and Fig. 2 (ARGs coding for resistance against gentamicin), the true occurrence of CPM, the CPM in a pooled sample, and the CPM measured using metagenomics is illustrated. In both figures, the observed occurrence is fluctuating around the truth as a result of both sampling error and laboratory noise. The effect of increasing the number of samples per pool, and at the same time reducing the sequencing depth in the metagenomics lowers the deviation between the true and observed data for both the highly abundant and low abundant genes.

Using the deviation from the truth with the sampling schedule of five samples per pool as a reference, the relative deviation from the truth when changing the sampling schedule (increased number of samples per pool and reduced sequencing depth) was calculated for monitoring the occurrence of ARGs coding for tetracycline efflux pumps (Table 1) and ARGs coding for gentamicin resistance (Table 2). Overall, the deviation from the truth when using 50–100 samples per pool is about 1/3 relative to the deviation from the truth when using 5 samples per pool.

**Table 1:** The relative deviation from the true occurrence of ARGs coding for tetracycline efflux pumps when increasing the number of samples from 5 (reference) to 20, 50, and 100 samples per pool per month (and subsequently reducing the sequencing depth); and the relative distribution of the absolute deviation due to the number of samples and sequencing depth.

# samples per pool per month	The relative size of the total deviation	Relative distribution of the deviation due to the number of samples/sequencing depth
5	100% (reference)	92%/8%
20	51%	85%/15%
50	33%	77%/23%
100	27%	61%/49%

**Table 2:** The relative deviation from the true occurrence of ARGs coding for resistance against gentamicin when increasing the number of samples from 5 (reference) to 20, 50, and 100 samples per pool per month (and subsequently reducing the sequencing depth); and the relative distribution of the absolute deviation due to the number of samples and sequencing depth.

# samples per pool per month	The relative size of the total deviation	Relative deviation due to sampling/lab
5	100% (reference)	93%/7%
20	51%	82%/18%
50	35%	71%/29%
100	29%	53%/47%

**Figure 3:**
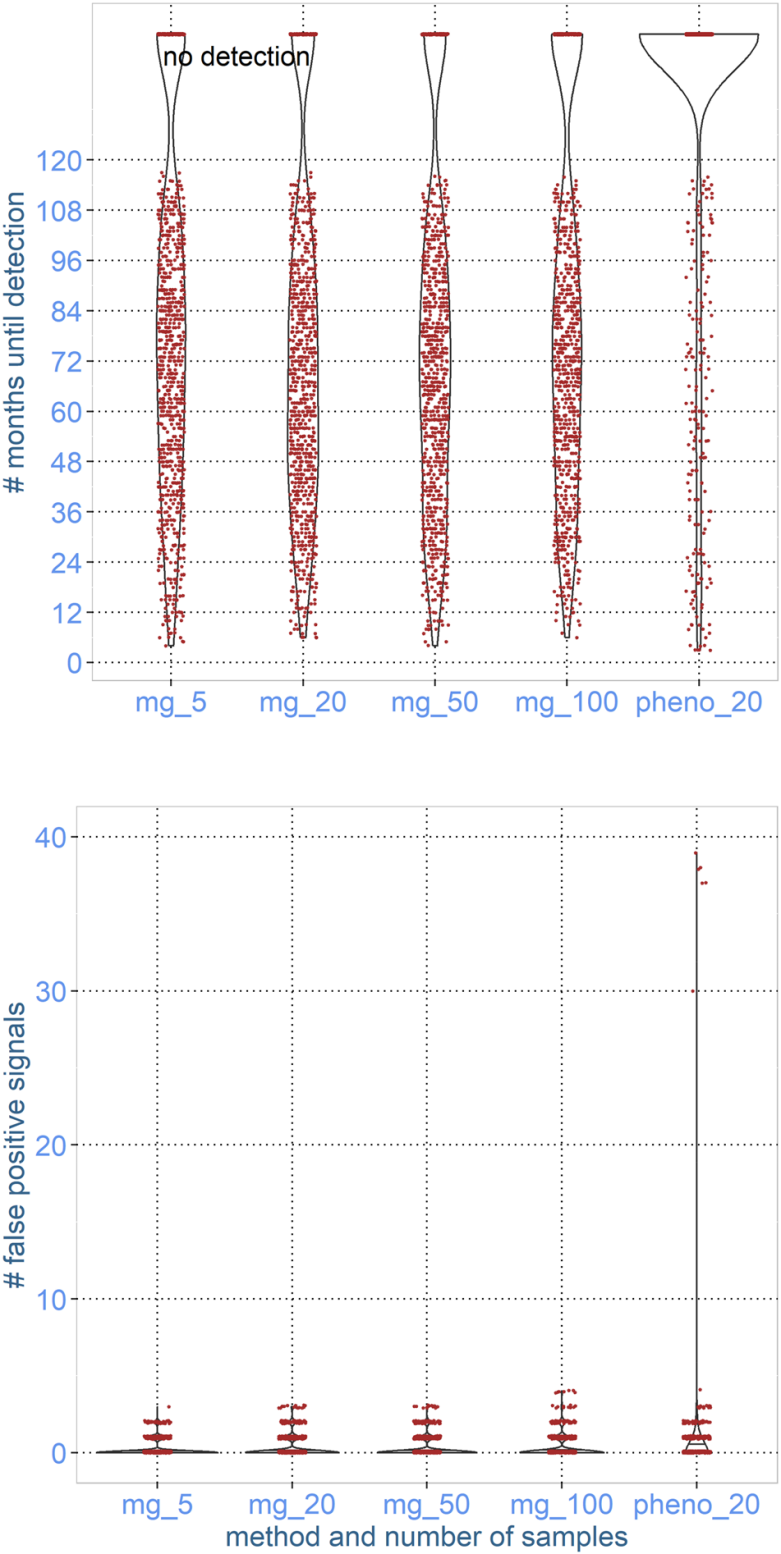
Results from simulation of monitoring significant changes in the occurrence of resistance against tetracycline in a pig population using 5, 20, 50, or 100 faecal samples per month. The occurrence of resistance is measured as the abundance of ARGs coding for efflux pumps causing resistance against tetracycline using metagenomics (mg) or phenotypic resistance in *E.coli* (pheno). The simulation was conducted on a scenario with no increase for 10 years (causing false negative signals—lower graph) and subsequently a 5% annual increase over 10 years (where we measured time to detection—upper graph).

**Figure 4:**
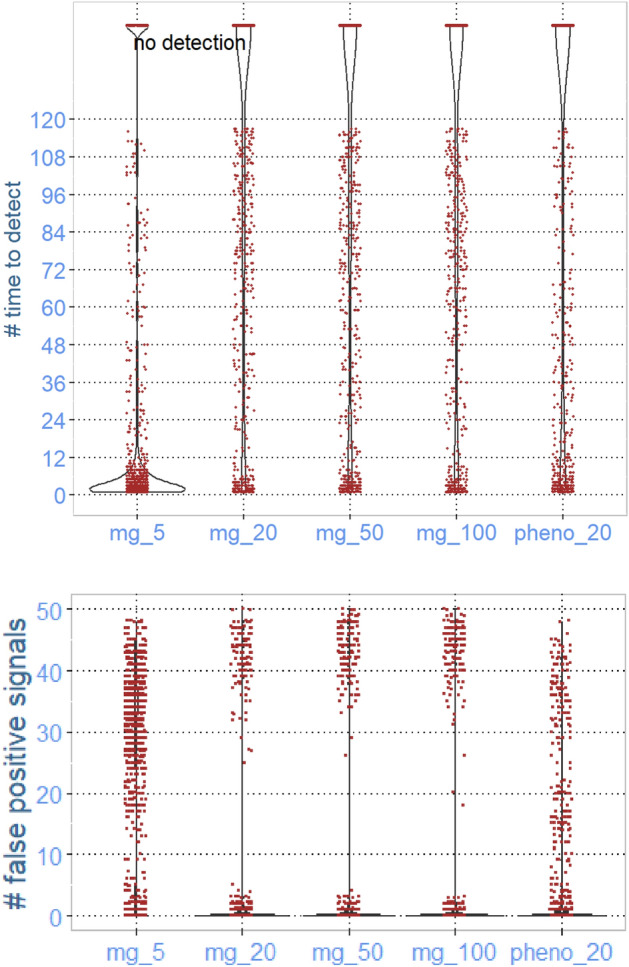
Results from simulation of monitoring significant changes in the occurrence of resistance against gentamicin in a pig population using 5, 20, 50, or 100 faecal samples per month. The occurrence of resistance is measured as the abundance of the ARG *aph* coding for resistance against gentamicine using metagenomics (mg) or phenotypic resistance in *E. faecalis* (pheno). The simulation was conducted on a scenario with no increase for 10 years (causing false negative signals—lower graph) and subsequently a 5% annual increase over 10 years (where we measured time to detection—upper graph).

In Figs. 3 and 4, the time until detection after the start of a true increase in each iteration using a sample size of 5, 20, 50, and 100 is presented using violin plots. Regardless of sample size, the range of the time to detect was from 1 month to not detected within 120 months, and there was no apparent difference in the time to detect between the sample sizes used in the monthly pool. In the simulated surveillance based on phenotypic characterisation of isolates, there were more iterations resulting in no detection within the first 120 months after the start of changes for common resistance, indicating that the amount and type of information (resistance/sensitive isolate) in the current monitoring system does not have the same power to detect changes in the occurrence of highly abundant resistance. In the simulation of phenotypic characterisation for detecting changes in the occurrence of low-abundant resistance, there was no apparent difference in the time to detect, wherever using metagenomics or phenotypic characterisation of isolates.

The number of false positive signals indicating a change in the occurrence of highly abundant ARGs was not dependent on the sample size (Fig. 3). The number of false signals for phenotypic characterisation of highly abundant resistance was of the same magnitude.

Opposite, the number of false positive signals indicating a change in the occurrence of low-abundant ARGs, was strongly dependent on the sample size (Fig. 4). In the 1001 iterations for 20, 50, and 100, respectively, there were zero false signals. When using 5 samples in the monthly pool, in more than 90% of the iterations, there were 10 false positive signals or more during the simulated time of 10 years with no true change. The number of false signals for phenotypic characterisation was of the same magnitude as in the scenario based on metagenomics of pooled samples with 20 or more samples per pool.

The more samples collected and pooled at each sampling point, the closer the sample represents the true occurrence of the ARG (Figs. 1 and 2). In the situation with 5 samples per pool, where a relatively large amount of the economical resources is spent on sequencing, the deviation in the observed data from the truth is due to the low representability of the sample because of the few animals in the pooled sample, and this cannot be compensated for by sequencing deeper. In Tables 1 and 2, the reduction in the overall deviation from the truth when increasing the number of animals per pool and a corresponding reduction in the sequencing depth is presented. Increasing the number of samples per pool from 5 to 50 or 100 will result in a deviation that is about 1/3 of the deviation when using 5 animals per pool.

In this study, we have used a statistical algorithm to analyse the simulated time series for significant changes in the observed data. We used the algorithm to summarise the simulated data and to generate patterns in the monitoring results in the different scenarios. We are not suggesting that this algorithm should be used in the monitoring program in real settings.

The algorithm was set to detect a change in trends over months, and not acute changes from 1 month to another. The outcome of interest in each time series was when a significant change was observed in the data during the 240-month long times series, and these time points were then compared with the defined truth.

In the two first scenarios, during the initial 120 months, there was no true change, so any significant time points detected in this time interval are false positive signals. In scenario 1, monitoring an ARG with relatively high abundance, the number of false negative signals in each time series ranged from 0 to 5 (Fig. 3) irrespective of the number of animals represented in the monthly pool. In the alternative monitoring approach, based on phenotyping 20 *E.coli* isolates per month, the number of false positive signals was similar, but with a tendency of more signals during the 120 months with no true change.

In scenario 2, monitoring an ARG with low abundance, due to the relatively large impact of selection error on the observed #CPM, there was a very large number of false positive signals (Fig. 4). In the sampling schedule using 5 samples per pool, it was almost random from month to month if there was a signal, and in most of the 1001 time series, there were more than 20 false positive signals. The number of false negative signals decreased with an increased number of samples in each pool. At 100 samples per pool, the most common number of false positive signals in the 1001 iterations was 0, or only a few, but in several iterations, there was a false negative signal more than twice a year (more than 20 signals in total) (Fig. 4). Also, in the alternative monitoring approach, based on phenotyping 20 *E. faecalis* isolates per month, the number of false positive signals was high (Fig. 4).

In the scenario monitoring changes in the abundance of high-abundance ARGs already present in the population, the time to detect a true change ranged from 5 to 120 months for all sampling schedules, and it was not strongly influenced by the number of samples per pool, indicating the information needed to detect a change is collected using only a few samples.

However, in the scenario monitoring changes in the abundance of a low-abundance ARG, using only 5 samples gave, in general, the shortest time to detect. This partly illogical result is due to the impact of random error on the observed results being very high in this scenario. This result is also the explanation for the high number of false positive signals, and this is independent of the truth. Therefore, the signal of a significant change shortly after the change has started is just random. Considering the monitoring of changes in low-abundance ARGs, taking the number of false positive signals into account, especially at a few samples per pool, the predictive value of a positive signal indicating a change is very low. At an increasing number of samples per pool (50–100), the positive predictive value of a signal indicating a change increases, and should be considered as truly positive.

**Figure 5:**
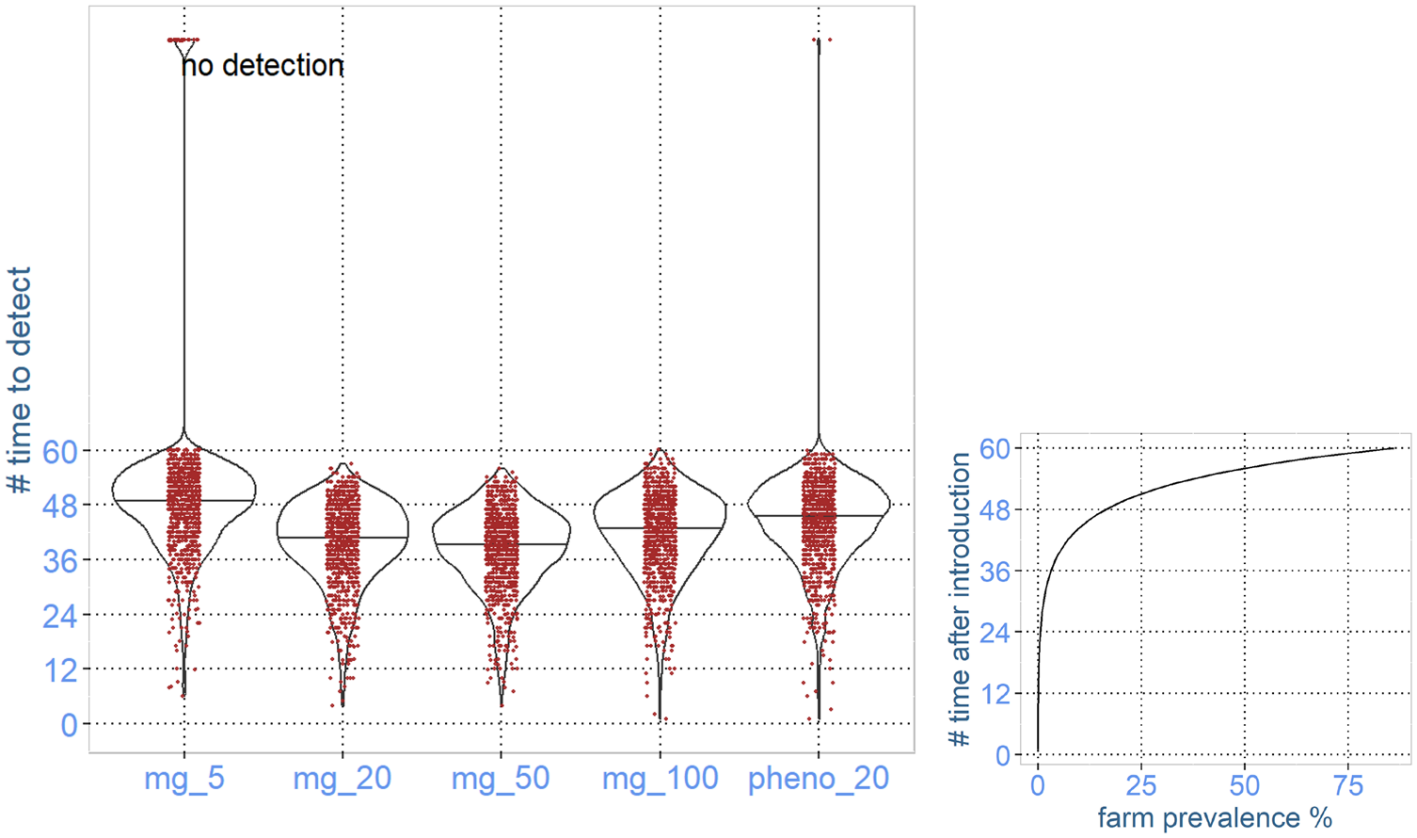
Results from 1001 iterations per sample schedule for time to detect a newly introduced AMR gene into the pig population. The simulated sampling schedules are a collection of faecal samples from 5, 20, 50, and 100 random pigs per month that are pooled into one technical sample before analysing with metagenomics (mg).

Figure 5 shows the distribution of time to detect the 1001 iterations done in each scenario with different numbers of samples in the pool. For any number of samples per pool, the time to detection varies by several months due to the stochasticity of sampling and laboratory, ranging from a detection within the first few months after increased occurrence to the increase detected first after several years. This variation in time to detection for any number of samples per pool was a result of the stochasticity of sampling from a population, and the randomness in which fragments that are sequenced and also partly by variation in the actual sequencing depth, which is not fixed.

The occurrence of the pathogen within animals on infected farms was set to the abundance of blaTEM in real farms.

In addition, the time to detection was also simulated using the phenotypic method, where the presence detected a new AMR strain of *E.coli*, with the same transmission rate between farms. The occurrence of the new strain in infected farms was set to the current prevalence of ESBL in pig production.

The right plot shows the prevalence of farms at different times after introduction (equivalent to the y-axis of the left graph) that was used in the simulation. The transmission rate is equivalent to that the gene is present in almost all farms 5 years after introduction into one farm at month 1 (prevalence 85%).

**Figure 6:**
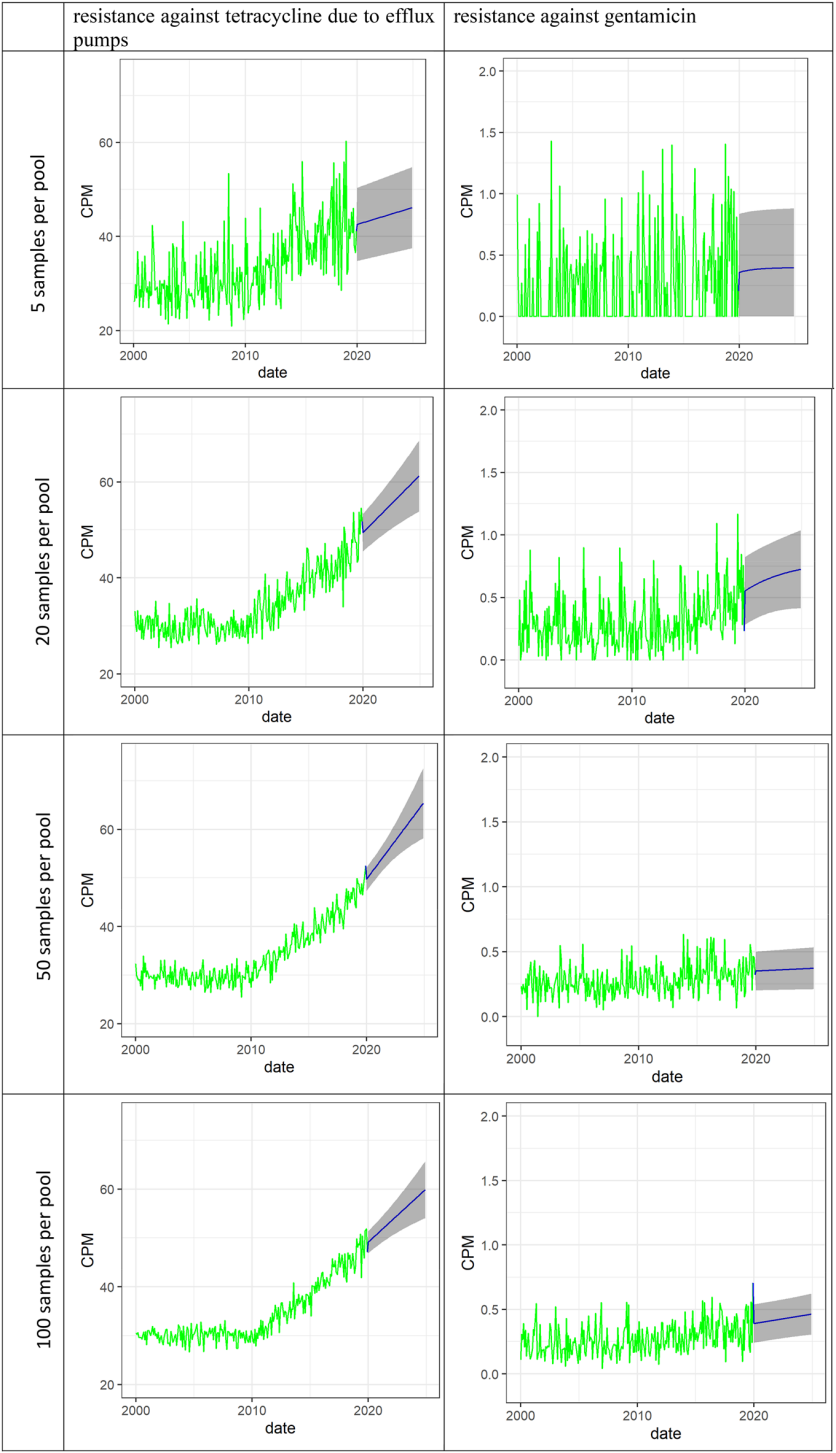
Plots of the forecasted trend of CPM of genes coding for resistance against tetracycline due to efflux pumps (left column) and genes coding resistance against gentamicin (right column). The green line show simulated observations obtained over 20 years of monitoring of resistance based on a monthly sampling of 5 (upper row), 20 (second upper row), 50 (third upper row), and 100 faecal samples (lower row) and metagenome analysis.

Figure 6 illustrates the 4-year forecast of the occurrence of an ARG based on the data that was observed in iterations in the period between 2000 and 2020. The size of the uncertainty in the forecast was increased by the period that was forecasted, but instead of an eyeball forecast, an analytical approach utilising the data is more valid, robust, and objective. The close approximation of the observed and forecasted data to the true data in Fig. 6 reveals that the performance of the surveillance is a valid representation of the truth and can be used to forecast the occurrence.

## Discussion

In this study, we modelled the utility of metagenomics in livestock production for detecting, monitoring, and forecasting the presence of ARGs. The model was developed on two dimensions: first, the dynamic change in the occurrence of AMR, and second, the monitoring procedure—sampling schedule, laboratory method, interpretation, and statistical analysis of laboratory results. Like all mathematical models, these results must be interpreted in the context of the model assumptions and the quality of available simulated data. Also, the results are based on ARG data from Danish pigs and assumptions about the spread of ARGs between farms in the Danish pig production system. The earlier indication of a change of AMR in a population, as more effective preventive measures will be (regulating or banning the use of certain antimicrobials) in respect to limiting the occurrence of AMR. However, to the best of our knowledge, this is the first mathematical model developed for estimating the performance of an AMR surveillance program based on metagenomics.

All of our simulations suggest a considerable time delay to detect either an increased occurrence of an ARG already present in the population (33–64 months) or the first detection of a completely novel ARG (19–33 months). However, our results also show that given the same amount of funding we might "gain" 14–31 months in time to detection by using the optimal pooling strategy combined with metagenomics. Also, as suggested by our simulations, the correct pooling strategy might be as effective as simply increasing the overall cost used for surveillance of AMR (in our case, 50%).

The generation of time-series data, in general, also gives the opportunity to do some kind of forecasting—either visually or analytically. In most surveillance programs related to health in animal or human populations, e.g., DANMAP^27^, the forecast is based on a visual inspection of the time-series data, aggregating the data to the annual occurrence. The use of metagenomics, in combination with a very strict sample schedule where samples are collected every month, will result in a time series about the quantitative amount of all known ARGs per, e.g., month. By estimating the trend in the observed data, the estimated trend can be used for a long-time forecast of AMR, given the trend is continuing. Given the potential of ARGs to transfer between bacteria species and the long time it takes to reduce the occurrence of AMR with preventive measures, forecasting the occurrence of AMR can support the implementation of preventive measures in due diligence, applying precautionary principles. This is especially relevant for AMR; decisions need to be taken before the adverse effects of AMR are observed in the human population.

The more sensitive we make the algorithm to detect changes, the greater the number of false alarms. The sensitivity of the whole surveillance must, in the end, be balanced against the size of the potential risk and the costs of false alarms, and in the end, that is a decision that needs to be made by risk managers. The parameter "duration" in the DBEST significantly detects change within the time series data. The shorter the "duration," the faster a breakpoint will be identified, but also, a short duration time will increase the likelihood of false alarms.

Compared to surveillance based on the phenotypic characterisation of isolated bacteria, the metagenomic method allows for monitoring the occurrence of all resistance genes with known nucleotide sequences in the same sample and laboratory procedure. Another strength is that the data structure of the outcome of metagenomics (#counts) makes it possible to aggregate the counts of each gene into higher levels, to obtain the #CPM of genes coding for resistance against antimicrobial class (tetracyclines, aminoglycosides, etc.). Thereby, surveillance of AMR based on metagenomics can reveal changes in the occurrence of AMR in the bacterial population at different levels. One apparent disadvantage of metagenomics is that the method does not indicate if the detected genes are phenotypically expressed in the population of bacteria. However, in the case of surveillance of AMR in populations of production animals, the risk is related to the likelihood that the gene is transferred to human pathogens. One of the deciding factors for the size of this risk is the absolute number of resistance genes in the microbiome. Therefore, in this context, whether the genes of interest are expressed or not in the collected sample is of minor concern.

The inherent benefit of using metagenomics to monitor AMR in a population is that it is possible to monitor the occurrence of all known ARGs within one technical sample. However, the power to detect changes in the occurrence of AMR depends strongly on the true occurrence of the AMR in the population and the criteria set defining a change. Traditionally, this can be handled by adjusting the sample size for each AMR of interest, so there is a given probability to detect a change in the monitoring system if it is present in reality. In monitoring based on metagenomics, this is not as straightforward, because we use the same sample for monitoring all known ARGs.

Within the range of the simulation settings in this study, also for monitoring based on metagenomics, the most important factor to obtain valid monitoring results is to have technical samples with a high representativeness of the population of interest. Compared to monitoring based on the characterisation of isolates, metagenomics can be applied to pooled samples. The results from our simulations indicate that, given the economical constraints, using technical samples of 50–100 pooled faecal samples give the most valid results when applying metagenomics for monitoring AMR in the Danish pig population.

Comparing the results of the scenarios with high and low abundance of ARGs, the metagenomics has the best performance in monitoring changes in highly abundant ARGs (faster to detect changes and fewer false signals). For monitoring changes in low-abundant ARGs, the performance is impaired by the fact that the random noise from sampling and laboratory procedure results in false positive signals in the observed data. The solution for this is to increase the magnitude of change that must be observed before it is classified as a change (increasing the cutoff value), but this will be at the cost of having a longer time to detect a true change. For the detection of new emerging ARGs, metagenomics has a better performance than phenotypic-based monitoring when using 50–100 samples per pool.

Before starting to use metagenomics to monitor AMR in a population, studies elucidating the effect of decisions about the criteria for classifying a change in observed data as a true change must be performed. The amount of data (and information) generated in metagenomics is comprehensively compared to the proportion of resistant isolates of pre-selected indicator bacteria species. The comprehensiveness of the sequencing data must be combined with robust approaches for summarising metagenomic data into outcome measures that are in alignment with the aim of the surveillance system.

Overall, the opportunity to succeed in strengthening the surveillance of AMR in animal populations using metagenomics is that this laboratory procedure can be applied to large pools of samples. Large pools of samples increase the representativeness of the monitored results. The dilution effect due to pooling can be handled by using a high sequencing depth. In addition, by using metagenomics, all known ARGs are included in the monitoring program.

In general, as a result of an increased sensitivity of monitoring, the likelihood of false signals also increases. The balance between sensitivity and specificity of a monitoring program is a decision more than a scientific question. The database we used in the metagenomic analysis to identify acquired ARGs currently contains about 5000 ARGs. Due to the magnitude of ARGs that can be monitored, a crude screening for significant changes will by chance result in hundreds of false alarms at every testing point of time.

In real settings, a well-functioning monitoring system is characterized by high negative and positive predicted values, which is the likelihood that the absence of signals in the monitoring system is a true reflection that there are no changes, and that a true positive monitoring signal is truly positive. A low negative predicted value in a monitoring system will result in the emergence of an increased occurrence of ARGs and it will be more extended before it is identified and preventive measures are implemented. A low positive predictive value in a monitoring system is not useful because the unsubstantiated or false-positive reports result in unnecessary investigations and wasteful allocation of resources.

Monitoring the changes in highly abundant ARGs, the outcome from the 1001 iterations using different numbers of samples and sequencing depth was similar, indicating that both positive and negative predictive values are the same across different numbers of samples per pool and sequencing depth. For the phenotypic characterisation of isolates to identify changes, the positive predicted was similar to what was found in the monitoring based on metagenomics, but the negative predicted value is significantly smaller, and in many iterations, the true change was not detected.

To detect a newly introduced AMR gene or isolate in the pig population, in the simulations settings, almost irrespectively of metagenomics or phenotypic detection, or the number of samples used in the pool, the AMR will be detected within 5 years (Fig. 5). Overall, the setup with 50 samples per monthly pool and using metagenomics gave the shortest time to detection. In a few iterations using 5 samples per pool and metagenomics, or phenotypic characterisation of 20 isolates monthly, a newly introduced AMR was not detected.

The presented results from our simulation demonstrated that the overall performance of a monitoring program of AMR in the Danish pig population depends on the abundance of AMR in the population, sampling schedule and laboratory method, how fast the occurrence of AMR is changing in the population and the threshold for classifying an observed change as a significant change.

In the situation of monitoring AMR in a population, the value of the positive and negative predicted value of the monitoring system is dependent on the characterisation of the monitoring system (sample schedule, data generated via the laboratory method, and algorithm to identify signals), and the true occurrence of AMR in the population that is monitored. To optimize the overall negative and positive predictive values of monitoring ARGs in a population, the algorithms used for screening the results must take into account which ARGs or groups of ARGs impose important risks to humans. In addition, a comparison of the consequences of false signals (unnecessary investigations and wasteful allocation of resources) and the consequence of time between the occurrence of change and detection (uncontrolled spread of the ARGs) should be considered when defining the threshold used for indicating a change in the occurrence.

The overall strength of using metagenomics compared to traditional phenotypic characterisation of isolates is that all known ARGs can be monitored within the same laboratory analysis. Also, the metagenomic approach makes it feasible to work with pooled samples, whereby a larger proportion of the population of interest can be represented in the sample.

## Methods

**Figure 7:**
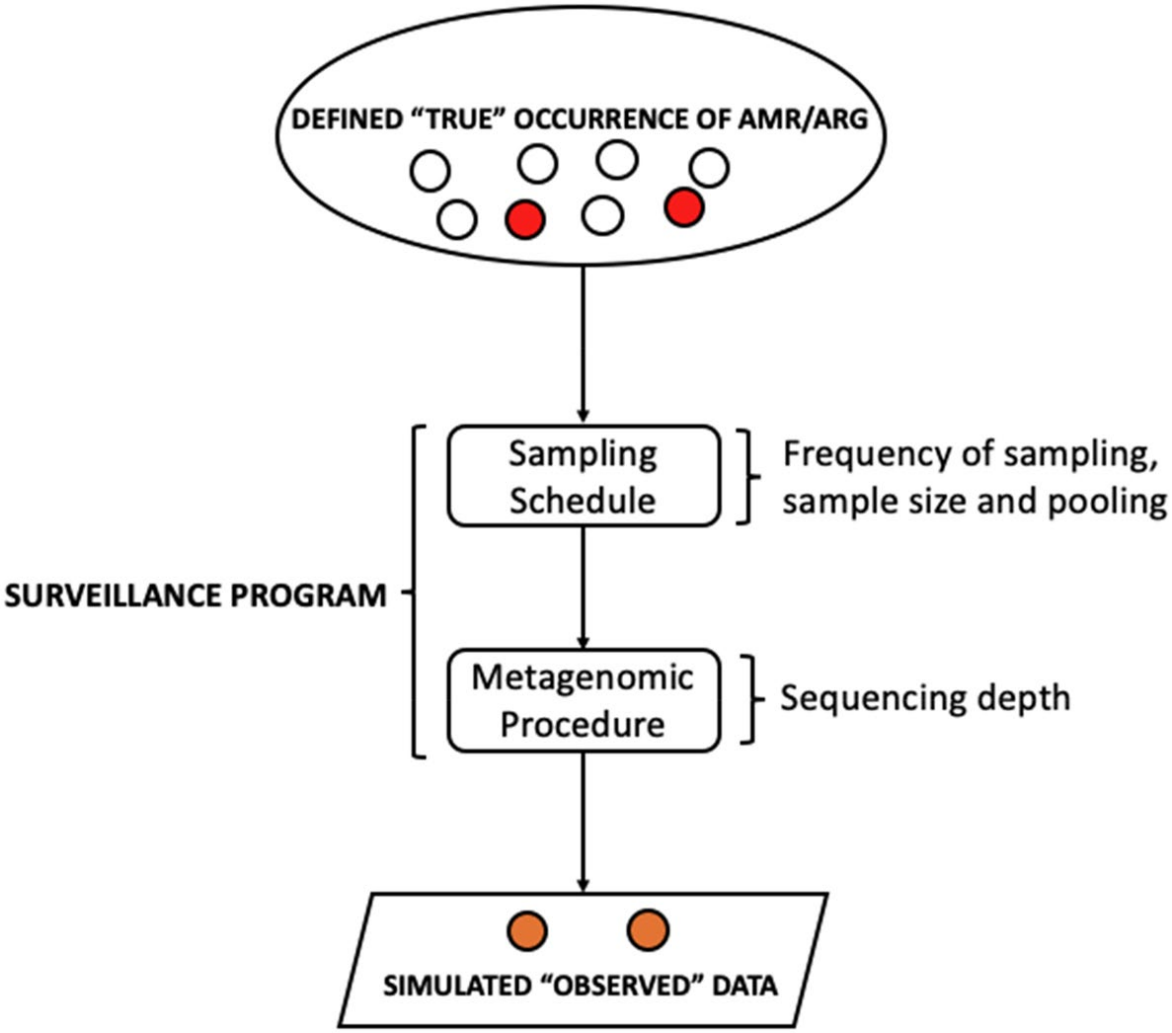
Study flowchart for study design adapted in this study.

The design of the study is presented in Fig. 7. Initially, we developed a dynamic mathematical model describing the occurrence and the change in the occurrence of ARGs of interest in the population over time. This model describes the "true" occurrence of ARGs of interest at any given time.

Secondly, a model mimicking the surveillance program overlaid the model of the "true" occurrence to simulate the results that can be observed in surveillance given the settings of the sampling schedule (frequency of sampling, sample size, and pooling) and the metagenomic procedure (sequencing depth). In each iteration, by taking into account the stochasticity of sampling and metagenomics, a time series of observed data was generated. Each time series was subsequently analysed statistically to identify time points for the detection of the start of changes in the occurrence as well as to estimate long-term trends. In addition, given the time series, the future occurrence of the ARG of interest in the population was forecasted using a forecasting methodology. The results from the statistical analysis were finally compared with the known truth that was given in the dynamic model describing occurrence and the change in the occurrence of an arbitrary ARG in the population over time.

### Mathematical notification of model describing "true" occurrence and the change in the occurrence of an arbitrary ARG in the population over time

True occurrence of an ARG in this study was defined as the occurrence of an ARG at the farm (proportion of farms positive), within farm prevalence (proportion of pigs within a positive farm with the ARG), and the concentration of the ARG in faeces of pigs carrying the gene. The concentration of the ARG(s) of interest in faeces was obtained from a lognormal distribution fitted to real-world data of the concentration of ARGs in pig faeces^28,29^. We chose a lognormal distribution because this gave the best fit to the data as compared with gamma and Weibull (for details, see Supplementary Fig. 1).

In this study, three different scenarios of true occurrence were modelled, each representing ARGs with different occurrences and changes: (1) an increased occurrence of an ARG that is already widely present in all farms in all pigs (high-level endemic), (2) an increased occurrence of an ARG that is present but at a lower frequency (low-level endemic) and (3) the de novo emergence of a novel ARG (emerging). In all scenarios, we used metagenomic data obtained from the samples collected in a previous study conducted on the Danish pig population^28^. In scenario 1, we used CPM of ARGs coding for efflux pumps causing resistance against tetracycline (tet(A), tet(B), tet(C), tet(G), tet(H), tet(K), tet(L), tet(Z), tetA(P), tet(30) and tet(40). This type of AMR is widely spread in the Danish pig population, and therefore in scenario 1, the proportion of pigs carrying this AMR was set to 100%, where the concertation was varied between pigs. Within the first 120 months, we assumed that there was no increase in concentration. After 120 months, we introduced a 5% annual increase in concentration.

In scenario 2, we used CPM of ARGs coding resistance against gentamicin by enzymatic modification (aac(6′)-Ie-*aph*(2″)-Ia)). This type of AMR rearely occurs in the Danish pig population, and therefore in scenario 2, the prevalence of animals within farms was set at 11%, in accordance with results from an observational study in Danish pig farms^28^. Within the first 120 months, we assumed that there was no increase in the prevalence of pigs carrying this AMR. After 120 months, we introduced a 5% annual increase in prevalence.

In scenario 3, new ARG was introduced into one farm in a population of 4,000 farms, where the ARG was allowed to spread to other farms over time (emerging). The spread was following a generalized Susceptible-Infected ($SI$) compartmental model^30^ (for details, see Supplementary Eq. 1). Our model was based on a homogeneous mixing system, with a transmission rate (0.148) resulting in 85% contaminated farms after 5 years. The spread described in the $(SI)$ model only considers the spread of ARG between farms. As soon as an ARG is introduced into a farm, it was assumed that the occurrence of pigs carrying the ARG was the same as the occurrence in a farm that has been infected for a longer period (10%). We assumed that the within-pig concentration of the emerging ARG was the same as the concentration of the blaTEM gene by results from an observational study in Danish pig farms^28^, where the CPM was extremely low.

To compare the use of metagenomics in surveillance with the current surveillance based on culturing, a model mimicking this surveillance was also developed based on the approach and results from the DANMAP surveillance. Phenotypic tetracycline resistance in the indicator bacteria Enterococcus faecalis [75% in 2021 (DANMAP 2021)] was used as a model for an increased occurrence of antibiotic resistance that is already widely present in all farms in all pigs (high-level endemic), phenotypic gentamicin resistance in the indicator bacteria *E.coli* (6% DANMAP 2021) was used as a model for an increased occurrence of antibiotic resistance that is present but at a lower frequency (low-level endemic). For the de novo emergence of novel antibiotic resistance, we assumed that the clone is introduced to one farm, and followed the same spread between farms as described for the spread of emerging ARGs (scenario 3). As soon as the clone is introduced into a farm, it was assumed that the occurrence of pigs carrying the clone was the same as the occurrence in a farm that had been infected for a longer period (90%). We assumed that the within-pig occurrence of the emerging clone was the same as the occurrence of phenotypic gentamicin resistance in the indicator bacteria *E.coli* (1% DANMAP 2021).

### Model for simulating results obtained in the surveillance program

In the simulation, we generated results obtained in a hypothetical monitoring program, including the effect of the design of the monitoring—the frequency of sampling and the number of samples at each timepoint of sampling. In this study, we assumed that all samples collected at each time point were pooled into one single technical sample before sequencing. Depending on the number of DNA fragments that are sequenced (sequencing depth)^31–33^ and the concentration of the ARG of interest^34^, the initial output from the model is the number of DNA fragments that were assigned to the ARG of interest.

Subsequently, we calculated the Counts of ARG divided by the total millions of fragments sequenced in the sample—Counts Per Million (#CPM)^35,36^. Thereby, the outcome of the surveillance of AMR using metagenomics is the #CPM in the population. The #CPM in each faecal sample is the product of whether the sample is collected from a farm having the ARG (0 or 1) $binomial\left( {n = 1, prob = farmprev} \right)$, the collected animal within a positive farm carrying the ARG (0 or 1) $binomial\left( {n = 1, prob = farmprev} \right)$ and the concentration of the ARG if the animal is carrying the ARG (random draw from the lognormal distribution). The model includes a pooling procedure where the number of collected faecal samples at each timepoint of sampling (assumed to be 5, 20, 50, or 100) are pooled into one technical sample before DNA sequencing. The mean of the CPM in the pooled faecal samples represents the "concentration of the ARG in the technical sample at a given point in time." The simulation was conducted over 240 months, with a sampling frequency of once per month.

In theory, when the design factors frequency of sampling, sample size, or the sequencing depth goes infinite, the sensitivity of the monitoring program will become 100%, and the lag time between change of occurrence and detection of change will be zero. To obtain realistic values of the design factors, we developed an equation wherein the price of sampling and sequencing were integrated with the variables sample size and sequencing depth, summing up to a given total annual cost of a monitoring program^37^. We developed a cost function (Eq. 1) to estimate how deep we would be able to sequence each pooled technical faecal sample given a sampling of *n* faecal samples (*n* = 5, 20, 50, and 100) randomly collected once a month. The cost was calculated using Eq. (1).

1 $$ \begin{aligned} Total price = & lab cost* k + lab technician cost \\ & \quad + n*price per sample + m*price per fragment \\ \end{aligned} $$

here $k$ is the number of technical samples processed in the lab per month (in this study $k=1$), and $n$ and $m$ are the number of samples in pooling and the sequencing depth, respectively (for details, see Supplementary Table 1).

An important determinant of the likelihood of metagenomics in detecting an emerging ARG is how many gene fragments are sequenced (sequencing depth)^38–40^. Fragments that are sequenced in a sample are random, whereas the number of sequenced fragments can be pre-defined within a certain range. The mean sequencing depth for each sample pool is determined using $rtruncnorm(n, a, b, mean, sd )$: where $n$ is the number of observations (months); $a$ is the lower mean sequencing depth; $b$ is the upper mean sequencing depth; $the mean$ is mean sequencing depth, and $sd$ is $(mean-a)/3$. To add randomness to the sequencing depth, we assume that the depth is varying stochastically between $\pm \hspace{0.17em}$20% of the mean, following a normal distribution where the range between -20% and + 20% is equal to 6SD. During simulation, the sequencing depth was truncated at $\pm \hspace{0.17em}$3SD^41^. In the simulation of all scenarios and sub-scenarios, 1001 iterations were performed using Monte Carlo simulation^42–44^, representing 1001 time series of the observations.

### Calculating deviations between observed and true occurrence

As given by the setup of the simulation, the deviation of the observed data from the truth originates from the sampling error at the collection of faecal samples (deviation due to sampling) as well as which gene fragments are sequenced (deviation due to lab procedure). These two sources of error are distinct from one another. To compare how these two sources of error contribute to the deviation between the observed data and the truth, for each observed data point in each time series, we decompose the total deviation into deviation due to sampling (Eq. 2) and lab procedure (Eq. 3)

2 $$ DeviationSampling\left[ {CPM} \right] = Sample\left[ {CPM} \right]{-} Truth\left[ {CPM} \right] $$

3 $$ DeviationLab \left[ {CPM} \right] = Observed\left[ {CMP} \right] - Sample\left[ {CPM} \right] $$

Subsequently, the absolute values of DeviationSampling and DeviationLab for each sampling schedule were summarised and compared relative to each other.

Also, to compare the overall effect of the number of samples and sequencing depth on how close to the truth the monitoring will be for each observed data point in each time series, the deviation is calculated according to Eq. (4)

4 $$ Deviation\_obs\_truth \left[ {CPM} \right] = Observed\left[ {CMP} \right] - True\left[ {CPM} \right] $$

Subsequently, the absolute values of *Deviation_obs_truth* were summarised and compared relatively between the different sampling schedules within scenarios one and two, respectively.

### Statistical analysis of the time series obtained in the simulations

The performance of the surveillance program^45^ for detecting ongoing changes in the occurrence of ARGs in the population was done by initially identifying a change in the observed data using breakpoint analysis^46,47^, and subsequently calculating the time between the start of the change given by the mathematical function defining the true occurrence of an ARG in the population, and the time point when this change was identified in the time-series of observed data.

By applying the breakpoint analysis throughout the time series of observed data, including the initial period of 10 years with no true change, we also obtained the number of false alarms, which is when the surveillance indicates a change in the occurrence of #CPM where the occurrence is not changing.

### Breakpoint analysis—DBEST method

The simulated observed data of an ARG in a population using metagenomics were analysed for the presence of changes in occurrence over time using breakpoint analysis. In reality, when the occurrence of endemic AMR starts to change, the increase/decrease takes place gradually over months/years and not with a direct shift from one level to another within a few months. In the R package DBEST, statistical methods are applied to analyse time trends and detect changes over time. DBEST was originally developed to both detect and forecast changes in vegetation using remote information^48,49^. We chose DBEST because of its ability to handle #CPM in the form of continuous data, which is not possible with surveillance package analysis programs that only consider whole numbers.

In our study, we used DBEST for time-trend analysis, looking for changes in the #CPM of the ARG(s) of interest over time (Supplementary Fig. 2). DBEST detects and estimates trend changes in two ways (abrupt and non-abrupt) and the timing, magnitude, number, and direction of those changes. In the DBEST method, five parameters must be defined before the analysis. The description of the "change_magnitude" is the size of changes in the data that indicate a potential true change. In this study, we used a 5% increase/decrease (change_magnitude, Table 1) from the #CPM mean of the ARG of interest in the population before an increase occurred. The specified "duration" is the period after the breakpoint that the change must be present to indicate a true persistent change, given the algorithm's sensitivity to detect changes. This was defined as 6 months in our study (Table 3).

**Table 3:** The parameters used for the DBEST method.

Parameter	Value
First-level-shift	*Reference_Mean
Second-level-shift	*Reference_Mean
Duration	6 months
Alpha	0.05
Change_magnitude	5% of *Reference_Mean

*Reference_Mean is the mean of the AMR gene of interest in the population before an increase takes place.

Following our knowledge of changes in ARGs in animal populations over time and how it was specified in the model, the statistical methods should identify non-abrupt changes over a longer period. Abrupt changes in a short time interval are not expected, and therefore, the parameters in the algorithm were set so it only looks for non-abrupt changes. Therefore, the values for "first-level-shift" and "second-level-shift" were set to extremely high values, whereby none of the detected change points were classified as abrupt. This is a technical issue, but it is worth noting. The "alpha" is the statistical significance level value used to test the significance of detected changes. Table 3 shows how these five parameters were implemented in the DBEST.

### Forecasting

In this study, based on the simulated data, we also tried to forecast the trend for 5 years (60 months) using the R package “forecast”. The trend was obtained using exponential smoothing assuming additive errors, additive trend, and no seasonality.

## Author contributors

O.O.A., F.M.A., and H.V. conceived the study. O.O.A. and H.V. designed the study. All authors acquired and analysed the data. O.O.A., F.M.A., and H.V. interpreted the findings. O.O.A. wrote the first draft of the manuscript. F.M.A. and H.V. contributed to the writing and subsequent versions of the manuscript. O.O.A. developed the code. All authors critically reviewed this paper and approved the final version. The corresponding author (O.O.A.) had final responsibility for the decision to submit for publication.

### Supplementary Information

Supplementary Information.

## Data Availability

The results from fitting probability distribution to counts per million for genes coding for tetracykline efflux pumps and gentamicin enzymatic modification is presented in the supplementary material of this article. Examples of output from the R package DBEST used to analyse the simulated surveillance data are presented in the supplementary material of this article. All data and codes used to generate the results presented in this publication is available in the surveillance simulation metagenomics repository at github (https://github.com/HakanVigre/surveillance_simulation_metagenomics/).
