# Supplementary material for: Modelling the effectiveness of surveillance based on metagenomics in detecting, monitoring, and forecasting antimicrobial resistance in livestock production under economic constraints
Source: Sci Rep. 2023 Nov 21;13:20410. doi: 10.1038/s41598-023-47754-w (PMC10663573; doi:10.1038/s41598-023-47754-w)
Supplement: Supplementary file 1 — Supplementary Information. [file 41598_2023_47754_MOESM1_ESM.docx]

**Supplementary material to:**

**Modelling the effectiveness of surveillance based on metagenomics in detecting, monitoring, and forecasting antimicrobial resistance in livestock production under economic constraints**

Ofosuhene O. Apenteng^1,2^, Frank M. Aarestrup^1^ and Håkan Vigre^1^

**Affiliation**

^1^Research Group for Genomic Epidemiology, National Food Institute, Technical University of Denmark, Kongens Lyngby, Denmark

^2^Section of Animal Welfare and Disease Control, Department of Veterinary and Animal Sciences, University of Copenhagen, Denmark.

The study was performed by first simulating emerge of AMR in a population over time – we utilised real metagenomics data from the Danish pig production, and emergence was simulated using epidemiological models such as Susceptible- Infected ($SI$) model^1^

$$\frac{dS}{dt}=-\beta\frac{S}{N}\sum_{i}^{n} I_{i}$$

$$\frac{dI_{1}}{dt}=-\beta\frac{S}{N}\sum_{i}^{n} I_{i}-\sigma nI_{1}$$

$$\frac{dI_{i}}{dt}=\sigma nI_{i-1}-\sigma nI_{1} \forall_{i}=2, . . . , n$$

where $n$ is the number of stages in the infected period, where the movement between compartments is multiplied by $\sigma$ to maintain the average infected period even when the number of compartments changes.

Given a total economical constraint of 60,000 € (annually). We calculated the estimated sequence depth based on the constraints given by available funds. The following information was used to calculate the maximum sequencing depth of samples in the three different scenarios by (Eq.1). The cost of a lab technician is 1,350€/month, the cost of a lab is 10.94€/month, the cost of slaughter is 25.3€/month, and the cost of a fragment is (0.07€$\times$300*)/1e6, where 300* is the length of the fragment. All costs were estimated based on the current Danish cost and our almost 30 years of experience running the DANMAP program.

The cost we calculated from:

$$Total price=lab cost* k+lab technician cost+n*price per sample+m*price per fragment (Eq. 1)$$

where $k$ is the number of technical samples processed in the lab per month (in this study $k=1$), $n$ and $m$ are the number of samples in pooling and the sequencing depth, respectively.

The calculated sequence depths from (Eq.1) for pools of 5, 20, and 100, respectively for 5,000€ monthly are shown in Table 1. We used the truncated normal distribution, which is defined in the same way as the normal distribution in the simulation, with a 20% decrease and increase to obtain a range to limit the distribution to a lower and upper bound.

**Table 1: Calculation results from Eq.1 for the estimated sequence depth**

| Individuals/pool | Sequence depth (60,000€) |
| --- | --- |
| 5 | 167 million |
| 20 | 149 million |
| 50 | 114 million |
| 100 | 54 million |

We chose the Lognormal distribution because this gave a good fit to the data as compared to gamma and weibull distributions, as shown in Figure 1.

| 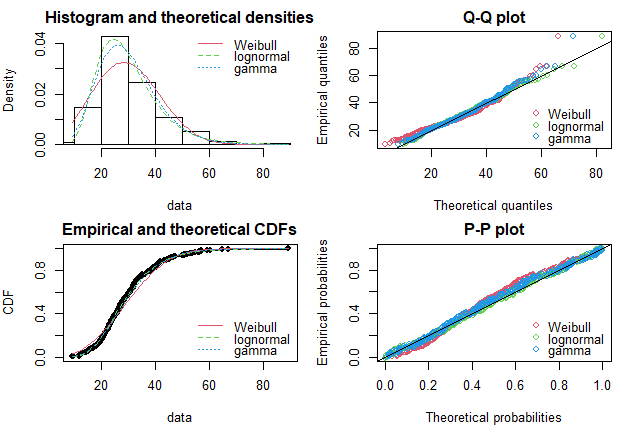   | 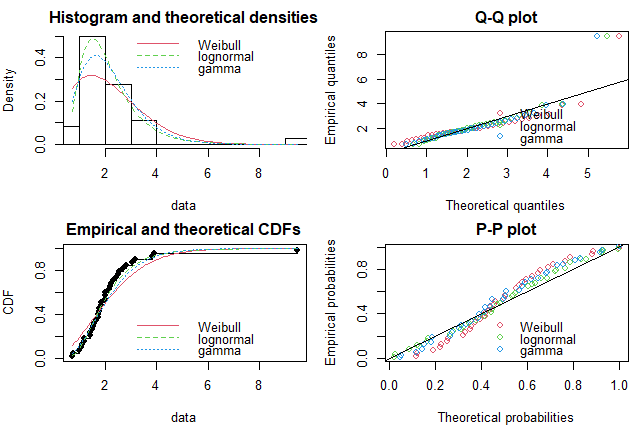   |
| --- | --- |
| **Figure 1:** Results from fitting lognormal, gamma and Weibull distribution to CPM data for tetracycline efflux genes (left column) and gentamicin resistance through enzymatic modification (right column). Lognormal distribution gave the best fit to the data as compared to gamma and weibull distributions | |

In the R package DBEST ^2^, statistical methods are applied to analyse time trends and detect changes over time, as shown in Figure 2. DBEST was originally developed to both detect and forecast changes in vegetation using remote information.

| 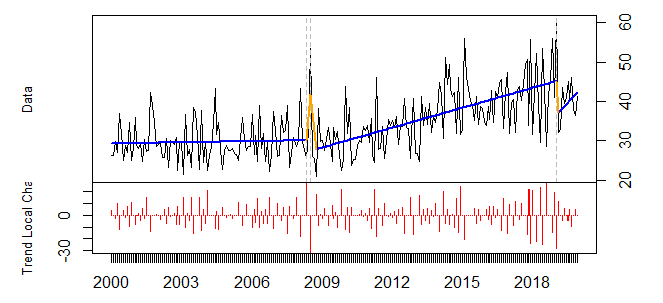  CPM_tetracycline (5 sample size) | 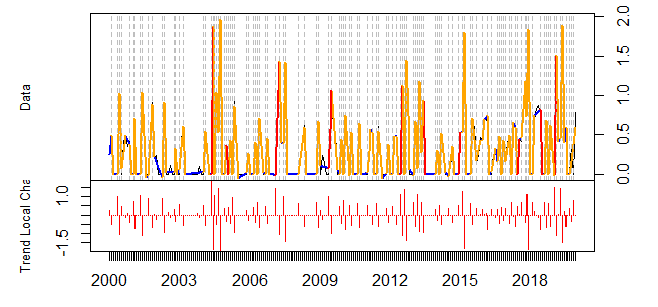  CPM_aph (5 sample size) |
| --- | --- |
| 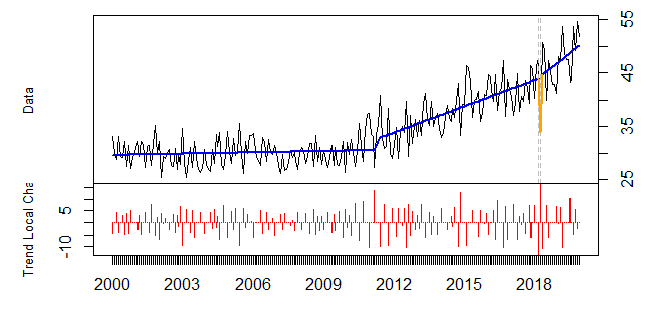  CPM_tetracycline (20 sample size) | 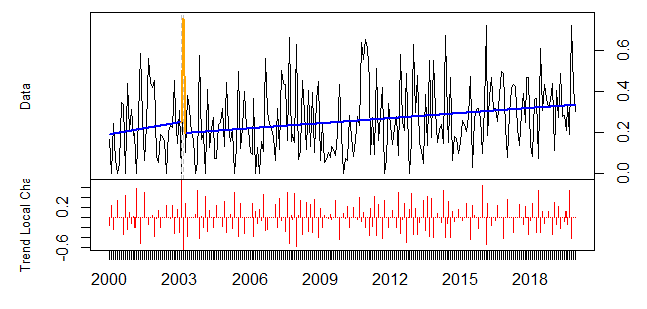CPM_aph (20 sample size) |
| 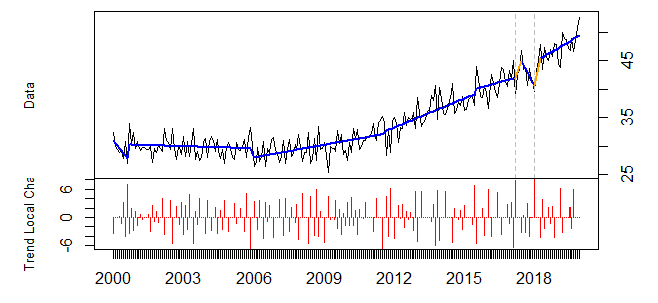  CPM_tetracycline (50 sample size) | 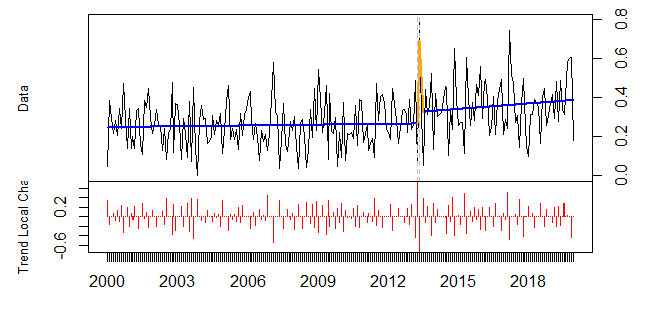CPM_aph (50 sample size) |
| 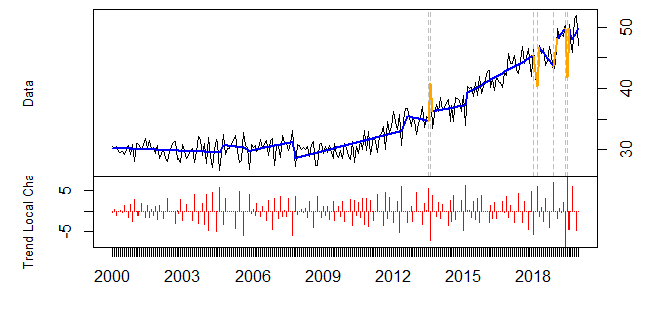  CPM_tetracycline (100 sample size) | 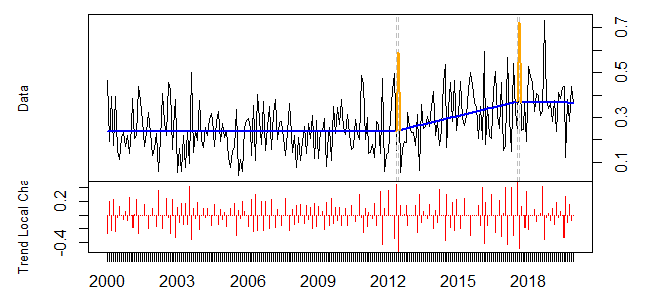CPM_aph (100 sample size) |
| **Figure 2: Results from surveillance of increased occurrence of AMR gene resistance. top – 5 samples per pool, middle 20 samples per pool, bottom 100 samples per pool.** Black: the observed data points. Blue: the fitted trend. Orange: the gradual changes detected. Dashed vertical lines mark the starting point of detected changes. | |

**Reference:**

1 Apenteng OO, Arnold ME, Vigre H. Using stochastic dynamic modelling to estimate the sensitivity of current and alternative surveillance program of Salmonella in conventional broiler production. *Sci Rep* 2020; **10**. DOI:10.1038/s41598-020-76514-3.

2 Jamali, S., Jönsson, P., Eklundh, L., Ardö, J. & Seaquist, J. Detecting changes in vegetation trends using time series segmentation. Remote Sens Environ 156, (2015).
